# The Long-Term Effects of Organophosphates Poisoning as a Risk Factor of CVDs: A Nationwide Population-Based Cohort Study

**DOI:** 10.1371/journal.pone.0137632

**Published:** 2015-09-04

**Authors:** Dong-Zong Hung, Hao-Jan Yang, Yu-Fen Li, Cheng-Li Lin, Shih-Yu Chang, Fung-Chang Sung, Sally C. W. Tai

**Affiliations:** 1 Department of Emergency, Toxicology Center, China Medical University Hospital, Taichung, Taiwan; 2 College of Medicine, China Medical University, Taichung, Taiwan; 3 School of Public Health, Chung Shan Medical University, Taichung, Taiwan; 4 Department of Family and Community Medicine, Chung Shan Medical University Hospital, Taichung, Taiwan; 5 Institute of Biostatistics, China Medical University, Taichung, Taiwan; 6 Management Office for Health Data, China Medical University Hospital, Taichung, Taiwan; 7 Graduate Institute of Clinical Medical Science and School of Medicine, College of Medicine, China Medical University, Taichung, Taiwan; Indiana University School of Medicine, UNITED STATES

## Abstract

**Background:**

Organophosphorus pesticides are widely used throughout the world. Because of their ease of availability, organophosphorus compounds are commonly used for self-poisoning in developing countries. The acute effects of exposure to organophosphorus pesticides are well known, but the chronic effects are unclear. Recent studies suggest that abnormalities of the central and peripheral nervous systems persisted for up to 5 years after acute poisoning due to a single large dose of organophosphates (OPs). However, the long-term effects on cardiovascular diseases are poorly understood.

**Methodology/Principal Findings:**

An OPs-exposed cohort (N = 7,561) and an age- and gender-matched control cohort (N = 30,244), both identified from the National Health Insurance Research Database, were compared. We utilized the multivariable Cox proportional model to estimate the risks of developing arrhythmia, coronary artery disease (CAD) and congestive heart failure (CHF). The patients with acute poisoning from OPs had higher incidence rates of arrhythmia (5.89 vs. 3.61 per 1,000 person-years), CAD (9.10 vs. 6.88 per 1,000 person-years), and CHF (3.89 vs. 2.98 per 1,000 person-years) compared with that of the non-OPs poisoning cohort, with a crude subhazard ratio (SHR) of 1.40, 1.13, and 1.12, respectively. Additionally, a significantly higher risk of arrhythmia was observed in the OPs poisoning cohort (adjusted SHR = 1.25) compared with the non-OPs poisoning cohort, particularly in male patients (adjusted SHR = 1.33) and those under 49 years of age (adjusted SHR = 3.16). After accounting for the competing risks of death, there was a higher risk of arrhythmia and CAD during a three year follow-up period (adjusted SHR = 1.50 for arrhythmia; adjusted SHR = 1.10 for CAD). We also found an adjusted SHR of 1.36 associated with developing CHF after 6 years of follow-up for OPs poisoning cohort.

**Conclusions:**

Acute OPs poisoning may continuously impact human health through mechanisms that are unclear. Any supportive measurements that could contribute to a reduction in the risk of heart disease may be beneficial in cases of OPs poisoning survivors.

## Introduction

Organophosphates (OPs) poisoning is an important public health problem in developing countries, because such compounds are widely used for the control of agricultural, industrial and domestic pests, and also leads to large numbers of cases of toxic effects on humans. Eddleston et al. indicated that organophosphorus pesticide self-poisoning kills an estimated 200,000 people every year [1]. Although most of such deaths occur in developing countries, this poisoning is also an important cause of fatal self-poisoning in developed countries [2]. Additionally, OPs can lead to a high potential of environmental pollution and increased health risks for the OPs poisoning patients who survive.

Acute OPs poisoning can cause acute cholinergic dysfunction, muscle weakness, seizures, coma, and respiratory failure. OPs stimulate both nicotinic and muscarinic acetylcholine receptors, as well as adrenergic receptors through the inhibition of acetylcholinesterase, which leads to the accumulation of acetylcholine and development of severe functional damage within both the central and peripheral nervous systems [1, 3]. Respiratory paralysis and cardiac arrest are considered to be the most common causes of death in acute OPs poisoning patients [4, 5]. Acute OPs poisoning is associated with three phases of cardiac manifestation. Firstly, there is a period of increased sympathetic tone, which is followed by a prolonged parasympathetic phase. Lastly, QT prolongation is followed by torsade de points ventricular tachycardia and ventricular fibrillation [6, 7].

Several studies have illustrated the long-term effects of OPs poisoning. Delayed polyneuropathy induced by OPs, but not carbamate, has been noted to be a harsh sequelae that results from exposure to certain OPs [8] and is characterized by distal degeneration of some axons of both the peripheral and central nervous systems occurring 1–4 weeks after single large dose acute exposures. Significant inhibition of neuropathy target esterases is potentially an etiological attribution of OPs-induced peripheral neuropathy. Few studies have also examined the long-term neuropsychiatric adverse effects of OPs after acute intoxication or multiple exposures to lower doses; however, those that have been conducted found increased occurrence of neurological or psychiatric presentations and poorer performance on standardized neuropsychological tests [9–12]. Few studies have illustrated an increase in the vulnerability of developing arrhythmias after accidental or terror-related OPs intoxication in experimental animals [13, 14]. No studies have demonstrated the long-term effects on the cardiovascular system after survival of OPs poisoning in humans, which is due to the difficulties in follow-up data collection.

Acute coronary syndrome and associated ventricular arrhythmia is one of the most prevalent causes of death in industrialized countries, as well as in developing countries [15, 16]. Despite a substantial amount being known and reported about classic cardiovascular risk factors such as family history, ethnicity, age, smoking, hypertension, dyslipidemia, diabetes, obesity, sedentary lifestyle, and dietary factors, there are still contentious areas that are not supported with conclusive evidence [17]. To improve the understanding of the long-term effects of OPs poisoning on cardiovascular diseases, we used a large population-based dataset to study the association between cardiovascular diseases (CVDs) and OPs poisoning. Our study provides explicit information on the relationship between OPs poisoning and CVDs, especially arrhythmia, coronary artery disease and congestive heart failure. This comprehensive investigation indicate that OPs poisoning could be a risk factor for the development of CVDs.

## Methods

### Data Source

This retrospective cohort study was conducted using Taiwan's National Health Insurance Research Database (NHIRD). The Taiwan National Health Insurance (NHI), a government-operated, universal health program established in 1995, has covered approximately 99% of the overall population and has been contracted by 97% of the hospitals and clinics nationwide. National Health Research Institute (NHRI) built and managed the NHIRD, which processed reimbursement claim data from the NHI program. The database contains comprehensive information on insured subjects, including dates of clinical visits, diagnostic codes, details about prescriptions, and expenditure amounts. Details of the database are presented on the NHRI website (http://www.nhi.gov.tw/english/index.aspx). Personal identifiers are encrypted for privacy protection, but all data sets can be linked to each other with the unique and anonymous identifiers created by NHRI. The present study was approved by the Institutional Review Board (IRB) of China Medical University and Hospital (CMU-REC-101-012).

#### Participants

The OPs poisoning cohort was identified from insured individuals with OPs poisoning (ICD-9-CM code 989.3) with an initial hospitalization between 2000 and 2011. The date of hospitalization diagnosis for OPs poisoning was designated as the index date. In both cohorts, individuals with a history of arrhythmia (ICD-9-CM code 427, 758.0, 758.1), coronary artery disease (CAD) (ICD-9-CM code 410–414), and congestive heart failure (CHF) (ICD-9-CM code 428) before the index date, or with incomplete age or sex information, were excluded. For each patient with OPs poisoning, four patients without OPs poisoning was randomly selected for the non-OPs poisoning cohort, using frequency matching method to insure both cohorts had same distributions over strata of sex, age (every 5-y span), and index year of OPs poisoning. Our further data analysis showed that the cumulative censoring rate over 12 years (2000–2011) was 24.0% in the OPs poisoning cohort, which was higher than that in the non-OPs poisoning cohort (10.0%). However, we did not have death records in the insurance data bases. The possible reasons for the discontinuity of national health insurance include death, withdrawal of insurance, immigration, prison sentence, etc.

#### Main Outcome and Co-morbidities

Each of the study subjects was followed until a diagnosis of arrhythmia, CAD, or CHF was made, until the patients were censored for loss to follow-up, death, withdrawal from the database, or the end of 2011, whichever event occurred first. We also incorporated inpatient diagnosis records to ascertain the baseline comorbidities, including diabetes (ICD-9-CM 250), hypertension (ICD-9-CM 401–405), hyperlipidemia (ICD-9-CM 272), and chronic obstructive pulmonary disease (COPD) (ICD-9-CM codes 490–492, 494, 496).

### Statistical Analysis

The distributions of demographic characteristics were compared between the OPs poisoning cohort and the non-OPs poisoning cohort. A Chi-square test was used for categorical variables, and a Mann-Whitney U test was used for continuous variables. Gender-, age- and comorbidity-specific incidences of each cardiovascular event were calculated for both cohorts. We used the Fine and Gray model [18], which extends the univariable and multivariable standard Cox proportional hazard regression model, to estimate the subhazard ratios (SHR) and 95% confidence interval (CI) for assessing the effects of OPs poisoning, the risk of CVDs for the OPs poisoning cohort, comparing with the non-OPs cohort, after accounting for the competing risks of death. The identification of death events was based on the discharge due to death, lost to follow up, or withdrew from insurance system in the NHIRD. The multivariable models were simultaneously adjusted for demographic status and co-morbidities of diabetes, hypertension, hyperlipidemia, and COPD. The Cox models were also used to evaluate whether OPs poisoning interacted with age (stratified ages into 2 levels ≤ 49 years and > 49 years) and with selected comorbidity (comorbidities into yes and no) in the association with the CVD. Additional analysis was conducted to assess whether the association between the risks of CVD and OP poisoning varied during the follow-up period. Risks of CVDs were estimated for three periods: ≤3 years, 4–6 years, and >6 years. We compared the Kaplan-Meier analyses to competing risk cumulative incidence of cardiovascular events between the OPs poisoning cohort and the non-OPs poisoning cohort using the Aalen-Johansen estimator [19]. All data analyses were conducted using SAS software (version 9.2 for Windows; SAS Institute Inc., Cary, NC, USA). A *P* value< .05 was considered statistically significant.

## Results

We selected 7,561 patients with OPs poisoning for our study cohort, and 30,244 control patients without OPs poisoning for our comparison cohort (Table 1). The median follow-up time of occurrence of arrhythmia were 5.94 (IQR = 3.96–9.63) years and 7.12 (IQR = 3.96–9.63) years in the OPs poisoning and non-OPs poisoning cohorts, respectively. The median follow-up duration for coronary artery disease was 5.81 (IQR = 2.10–9.03) years for the OPs poisoning cohort and 7.04 (IQR = 3.79–9.54) years for the non-OPs poisoning cohort. The median follow-up period for congestive heart failure was 6.07 (IQR = 2.23–9.15) years for the OPs poisoning cohort and 7.21 (IQR = 3.99–9.63) years for the non-OPs poisoning cohort.

**Table 1:** Number of events of OPs poisoning.

	OPs poisoning	
Year	No	Yes
2000	3844	961
2001	3896	974
2002	3840	960
2003	3420	855
2004	2888	722
2005	2596	649
2006	2308	577
2007	1884	471
2008	1612	403
2009	1452	363
2010	1308	327
2011	1196	299
Total	30244	7561

Among the study participants, 51.3% were older than 50 years of age, and 70.9% were men. The median age (IQR) of the OPs poisoning cohort and the non-OPs poisoning cohort were 50.5 (IQR = 39.0–63.6) years old and 50.3 (IQR = 38.6–62.9) years old, respectively. Compared with the non-OPs poisoning cohort, co-morbidity of diabetes (10.6% vs. 2.91%, p<0.001), hypertension (14.4% vs. 4.05%, p<0.001), hyperlipidemia (3.74% vs. 0.89%, p<0.001) and COPD (4.46% vs. 1.32%, p<0.001) were more common in the OPs poisoning cohort (Table 2).

**Table 2:** Characteristics of patients with OPs poisoning and matched patients without OPs poisoning.

	OPs poisoning				
	Yes (N = 7561)		No (N = 30244)		
	n	%	n	%	p-value
**Age, year**					0.99
≤34	1313	17.4	5252	17.4	
35–49	2372	31.4	9488	31.4	
50–64	2202	29.1	8808	29.1	
≥ 65	1674	22.1	6696	22.1	
Median (IQR) ^#^	50.5	39.0–63.6	50.3	38.6–62.9	0.02
**Gender**					0.99
Female	2202	29.1	8808	29.1	
Male	5359	70.9	21436	70.9	
**Comorbidity**					
Diabetes	803	10.6	881	2.91	<0.001
Hypertension	1085	14.4	1224	4.05	<0.001
Hyperlipidemia	283	3.74	269	0.89	<0.001
COPD	337	4.46	400	1.32	<0.001

Chi-square test

^#^:Mann-Whitney U test

IQR denotes interquartile range

Age means age at OPs poisoning.

Table 3 shows the incidence densities and subhazard ratios of multiple cardiovascular events by gender, age and co-morbidity. Overall, the patients with OPs poisoning had higher incidence rates of arrhythmia (5.89 vs. 3.61 per 1,000 person-years), and CAD (9.10 vs. 6.88 per 1,000 person-years), than the non-OPs poisoning cohort, with a crude SHR of 1.40 (95% CI = 1.21–1.61), and 1.13 (95% CI = 1.01–1.27). However, the association between OPs poisoning and CHF was not statistically significant.

**Table 3:** The risk of arrhythmia, coronary artery disease (CAD), and congestive heart failure (CHF) in patients and subhazard ratio for patients with OPs poisoning compared to patients without OPs poisoning by age, sex, and comorbidity in the competing-risk regression model.

			Overall	Gender^1^		Age, year^2^			Comorbidity^§^ ^3^	
				Female	Male	≤49	50–64	≥ 65	No	Yes
**Arrhythmia**										
OPs poisoning		Event	253	63	190	59	81	113	168	85
		PY	42980	12727	30253	23132	12612	7236	33965	9015
		Rate	5.89	4.95	6.28	2.55	6.42	15.6	4.95	9.43
Non-OPs poisoning		Event	734	207	527	65	201	468	571	163
		PY	203190	59941	143249	105035	60291	37864	192296	10894
		Rate	3.61	3.45	3.68	0.62	3.33	12.4	2.97	15
Crude SHR^†^(95%)			1.40(1.21, 1.61)***	1.23(0.93, 1.63)	1.46(1.24, 1.73)***	3.69(2.59, 5.25)***	1.64(1.26, 2.12)***	0.99(0.80, 1.21)	1.46(1.23, 1.74)***	0.55(0.42, 0.71)***
Adjusted SHR ^‡^(95%)			1.25(1.07, 1.46)***	1.02(0.75, 1.39)	1.33(1.12, 1.59)***	3.16(2.18, 4.59)***	1.32(0.98, 1.78)	0.89(0.72, 1.11)	1.59(1.33, 1.89)***	0.66(0.51, 1.00)
**CAD**										
OPs poisoning		Event	387	105	282	77	143	167	208	179
		PY	42536	12561	29976	23072	12354	7111	33792	8743
		Rate	9.1	8.36	9.41	3.34	11.6	23.5	6.16	20.5
Non-OPs poisoning		Event	1380	310	1070	161	493	726	1096	284
		PY	200499	59435	141065	104691	59056	36751	190034	10464
		Rate	6.88	5.22	7.59	1.54	8.35	19.8	5.77	27.1
Crude SHR^†^(95%)			1.13(1.01, 1.27)*	1.37(1.10, 1.72)**	1.06(0.93, 1.21)	1.94(1.47, 2.54)***	1.17(0.97, 1.41)	0.92(0.79, 1.10)	0.93(0.81, 1.08)	0.65(0.54, 0.79)**
Adjusted SHR ^‡^(95%)			0.96(0.85, 1.08)	1.10(0.86, 1.40)	0.92(0.80, 1.05)	1.48(1.10, 1.99)***	0.97(0.79, 1.19)	0.79(0.66, 1.00)	0.99(0.85, 1.15)	0.78(0.64, 1.00)
**CHF**										
OPs poisoning		Event	169	58	111	33	44	92	79	90
		PY	43427	12795	30632	23293	12770	7363	34339	9088
		Rate	3.89	4.53	3.62	1.42	3.45	12.5	2.3	9.9
Non-OPs poisoning		Event	607	182	425	41	151	415	441	166
		PY	203748	60124	143624	105154	60429	38166	192809	10939
		Rate	2.98	3.03	2.96	0.39	2.5	10.9	2.29	15.2
Crude SHR^†^(95%)			1.12(0.95, 1.33)	1.29(0.96, 1.73)	1.05(0.85, 1.29)	3.25(2.06, 5.14)***	1.17(0.84, 1.64)	0.90(0.72, 1.13)	0.88(0.69, 1.12)	0.56(0.43, 0.73)***
Adjusted SHR ^‡^(95%)			0.91(0.76, 1.09)	0.96(0.70, 1.32)	0.89(0.71, 1.10)	2.50(1.52, 4.10)***	0.79(0.54, 1.15)	0.78(0.62, 1.01)	0.97(0.76, 1.23)	0.70(0.54, 1.00)

PY, person-years; Rate, incidence rate per 1000 person-years; Crude SHR^†^, relative subhazard ratio; Adjusted SHR^‡^, subhazard ratio adjusted for age, sex, and comorbidities of diabetes, hypertension, hyperlipidemia and COPD; Comorbidity^§^: Patients with any one of the comorbidities diabetes, hypertension, diabetes, hyperlipidemia and COPD were classified as the comorbidity group;

*p<0.05

**p<0.01

***P<0.001.

^1^Adjusted SHR was calculated by competing-risk regression model stratified by gender and adjusted for age, and comorbidities of diabetes, hypertension, hyperlipidemia and COPD.

^2^Adjusted SHR was calculated by competing-risk regression model stratified by age, and adjusted for sex, and comorbidities of diabetes, hypertension, hyperlipidemia and COPD.

^3^Adjusted SHR was calculated by competing-risk regression model stratified by comorbidity and adjusted for age, and sex.

Multivariable competing-risks regression models for the risk of arrhythmia showed a significantly higher risk in the OPs poisoning cohort (Adjusted SHR = 1.25, 95% CI = 1.07–1.39) compared with the non-OPs poisoning cohort. The risk of arrhythmia was higher in men with OPs poisoning than in men without OPs poisoning (Adjusted SHR = 1.33, 95% CI = 1.12–1.39). The age-specific risk analyses showed that patients with OPs poisoning aged ≤ 49 years exhibited a significantly higher risk of arrhythmia than patients aged ≤ 49 years without OPs poisoning (adjusted SHR = 3.16; 95% CI, 2.18–4.59). In patients without co-morbidity, the risk of arrhythmia was 1.59-fold higher in the OPs poisoning cohort than in the non-OPs poisoning cohort (95%CI = 1.33–1.89). The age-specific analysis indicated that the OPs poisoning cohort exhibited a significantly higher risk of CAD than did the non-OPs poisoning cohort in ≤49-year age group (adjusted SHR = 1.48, 95% CI = 1.10–1.99). The younger patient group had a adjusted SHR of 2.50 developing CHF (95% CI = 1.52–4.10) in the OPs poisoning cohort compared to the non-OPs poisoning cohort.

In this study, the interaction measures between OPs poisoning and age, and between OPs poisoning and comorbidity associating with developing arrhythmia, CAD, and CFH (Table 4). Compared with patients ≤49 years and without OPs poisoning, patients >49 years and without OPs poisoning were associated with an increased risk of arrhythmia (adjusted SHR = 9.93, 95% CI, 7.69–12.8), followed by patients >49 years and with OPs poisoning (adjusted SHR = 9.64, 95% CI, 7.17–13.0) and patients ≤49 years and with OPs poisoning (adjusted SHR = 3.45, 95% CI, 2.43–4.91; interaction *P* < .001). Furthermore, relative to the non-OPs poisoning cohort without any comorbidity, the patients with any comorbidity were at a much higher risk of arrhythmia (adjusted SHR = 3.04, 95% CI = 2.11–4.37), followed by OPs poisoning patients and with comorbidity (adjusted SHR = 1.67, 95% CI, 1.13–2.46) and OPs poisoning patients and with any comorbidity (adjusted SHR = 1.46, 95% CI, 1.13–2.46; interaction *P* < .001). The risk of coronary artery disease and congestive heart failure had same trend which the effect between OPs poisoning and age or comorbidity. Though risk factors of age and comorbidity were important effect in CVD, the OPs poisoning effect to CVD still had statistically significant.

**Table 4:** Competing-risk regression model for the risk of coronary artery disease (CAD), and congestive heart failure (CHF) associated OPs poisoning with interaction of comorbidity.

	Age ≤ 49		Age > 49		Non-comorbidity				Comorbidity^§^	
	Non-OPs poisoning	OPs poisoning	Non-OPs poisoning	OPs poisoning	Non-OPs poisoning			OPs poisoning	Non-OPs poisoning	OPs poisoning
**Arrhythmia**										
Number	14740	3685	15504	3878		28135	5640		2109	1921
Event	65	59	669	194		571	168		163	85
Adjusted SHR^†^	1(Reference)^1^	3.45(2.43, 4.91)***	9.93(7.69, 12.8)***	9.64(7.17, 13.0)***		1(Reference)^2^	1.46(1.23, 1.74)***		3.04(2.11, 4.37)***	1.67(1.13, 2.46)***
p-value^&^	<0.001					<0.001				
**CAD**										
Number	14740	3685	15504	3878		28135	5640		2109	1921
Event	161	77	1219	310		1096	208		284	179
Adjusted SHR†	1(Reference)^1^	1.78(1.35, 2.33)***	7.25(6.14, 8.55)***	5.87(4.80, 7.19)***		1(Reference)^2^	0.99(0.85, 1.15)		2.06(1.79, 2.37)***	1.78(1.51,2 .10)***
p-value^&^	<0.001					0.004				
**CHF**										
Number	14740	3685	15504	3878		28135	5640		2109	1921
Event	41	33	566	136		441	79		166	90
Adjusted SHR†	1(Reference)^1^	2.86(1.81, 4.53)***	12.6(9.18, 17.4)***	8.89(6.17, 12.8)***		1(Reference)^2^	0.96(0.76, 1.23)		2.53(2.08, 3.07)***	2.00(1.58, 2.53)***
p-value^&^	<0.001					0.01				

Adjusted SHR^†^:adjusted for age and sex.

^&^p-value for interaction;

*p<0.05

**p<0.01

***P<0.001.

Comorbidity^§^: Patients with any one of the comorbidities diabetes, hypertension, diabetes, hyperlipidemia and COPD were classified as the comorbidity group.

^1^Adjusted SHR was calculated by competing-risk regression model adjusted for sex, and comorbidities of diabetes, hypertension, hyperlipidemia and COPD.

^2^Adjusted SHR was calculated by competing-risk regression model adjusted for age and sex.

Furthermore, the adjusted subhazard ratio varied during the length of follow-up period after OPs poisoning diagnosed (Table 5). The adjusted SHR for arrhythmia was significant in different follow-up durations (≤ 3 years, SHR = 1.50, 95% CI = 1.18–3.92; > 6 years, SHR = 1.40, 95% CI = 1.04–1.89). The incidence density rates of CHF increased with increasing follow-up periods in both cohorts. We found a 1.36-fold significantly higher relative risk of developing CHF after a 6 year follow-up period (95% CI = 1.01–1.84).

**Table 5:** Trends of cardiovascular event risks by stratified follow-up years in the competing-risk regression model.

	OPs poisoning							
	Yes			No				
Follow time, years	Event	PY	Rate	Event	PY	Rate	Crude SHR^†^ (95% CI)	Adjusted SHR^‡^ (95% CI)
**Arrhythmia**								
≤3	109	18017	6.05	250	82139	3.04	1.76(1.41, 2.21)***	1.50(1.18,3.92)**
4–6	80	13580	5.89	251	64485	3.89	1.26(0.97, 1.62)	1.13(0.87, 1.47)
>6	64	11383	5.62	233	56566	4.12	1.34(1.02, 1.77)*	1.40(1.04, 1.89)*
**CAD**								
≤3	178	17954	9.91	536	81712	6.56	1.34(1.13, 1.59)**	1.10(0.93, 1.31)
4–6	119	13406	8.88	464	63496	7.31	1.01(0.83, 1.24)	0.91(0.74, 1.12)
>6	90	11176	8.05	380	55290	6.87	1.15(0.91, 1.45)	1.06(0.83, 1.34)
**CHF**								
≤3	63	18111	3.48	203	82210	2.47	1.25(0.94, 1.66)	1.02(0.76, 1.37)
4–6	45	13744	3.27	211	64653	3.26	0.84(0.61, 1.16)	0.70(0.50, 1.00)
>6	61	11572	5.27	193	56886	3.39	1.53(1.15, 2.04)**	1.36(1.01, 1.84)*

PY, person-years; Rate, incidence rate per 1,000 person-years; Crude SHR^†^, relative subhazard ratio;

Adjusted SHR^‡^, subhazard ratio adjusted for age, sex, and comorbidities of diabetes, hypertension, hyperlipidemia and COPD.

*p<0.05

**p<0.01

***P<0.001.

**Fig 1:**
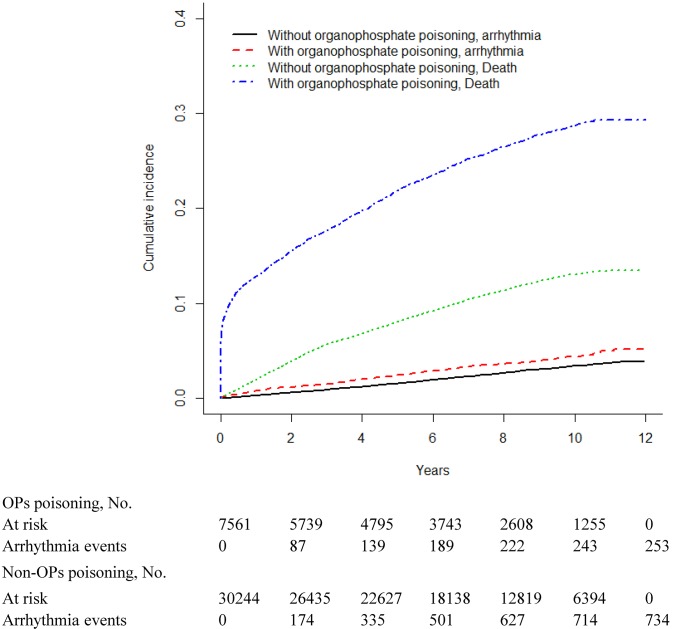
Cumulative incidence of arrhythmia compared between with and without organophosphate poisoning.

**Fig 2:**
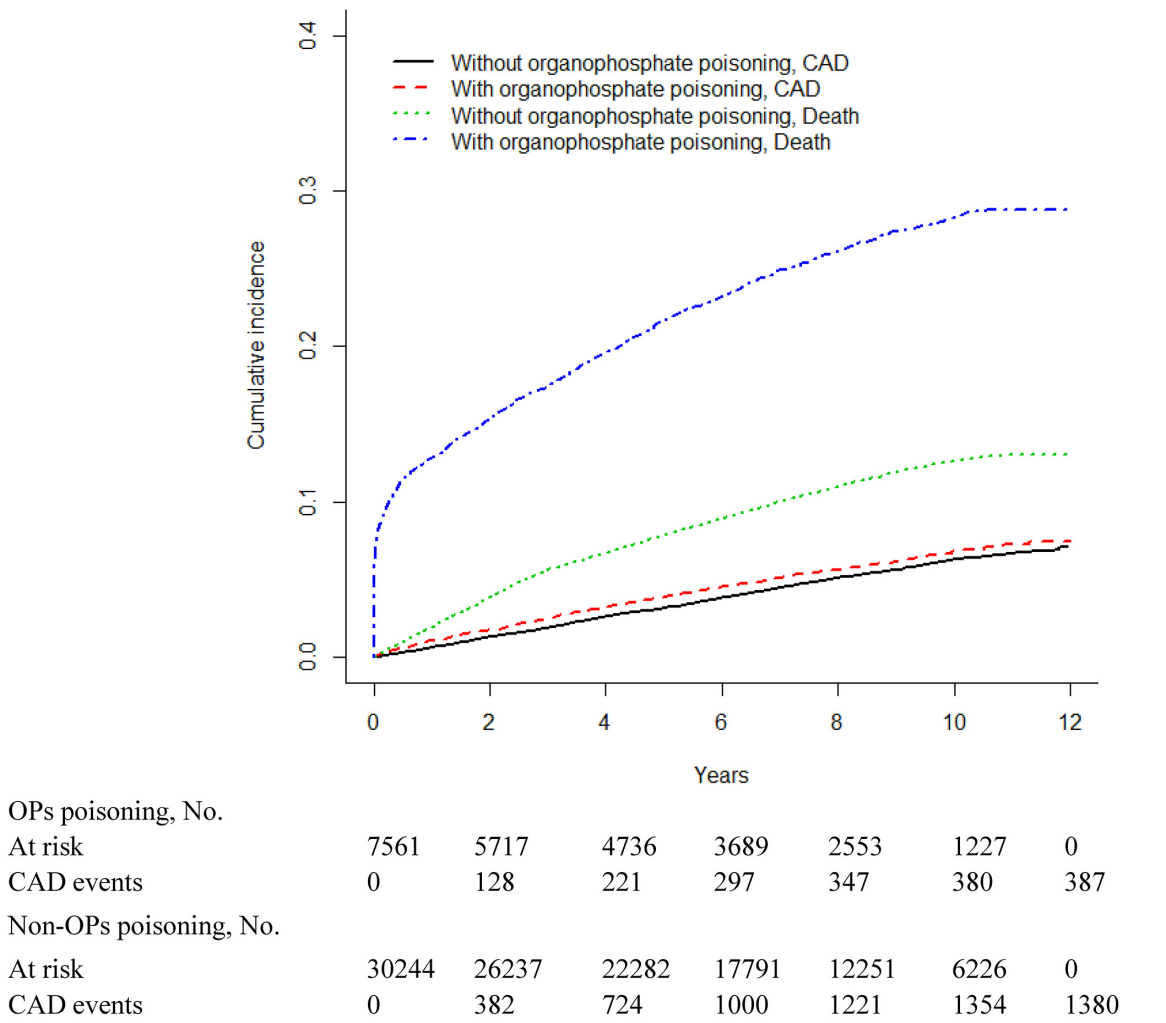
Cumulative incidence of coronary artery disease (CAD) compared between with and without organophosphate poisoning.

**Fig 3:**
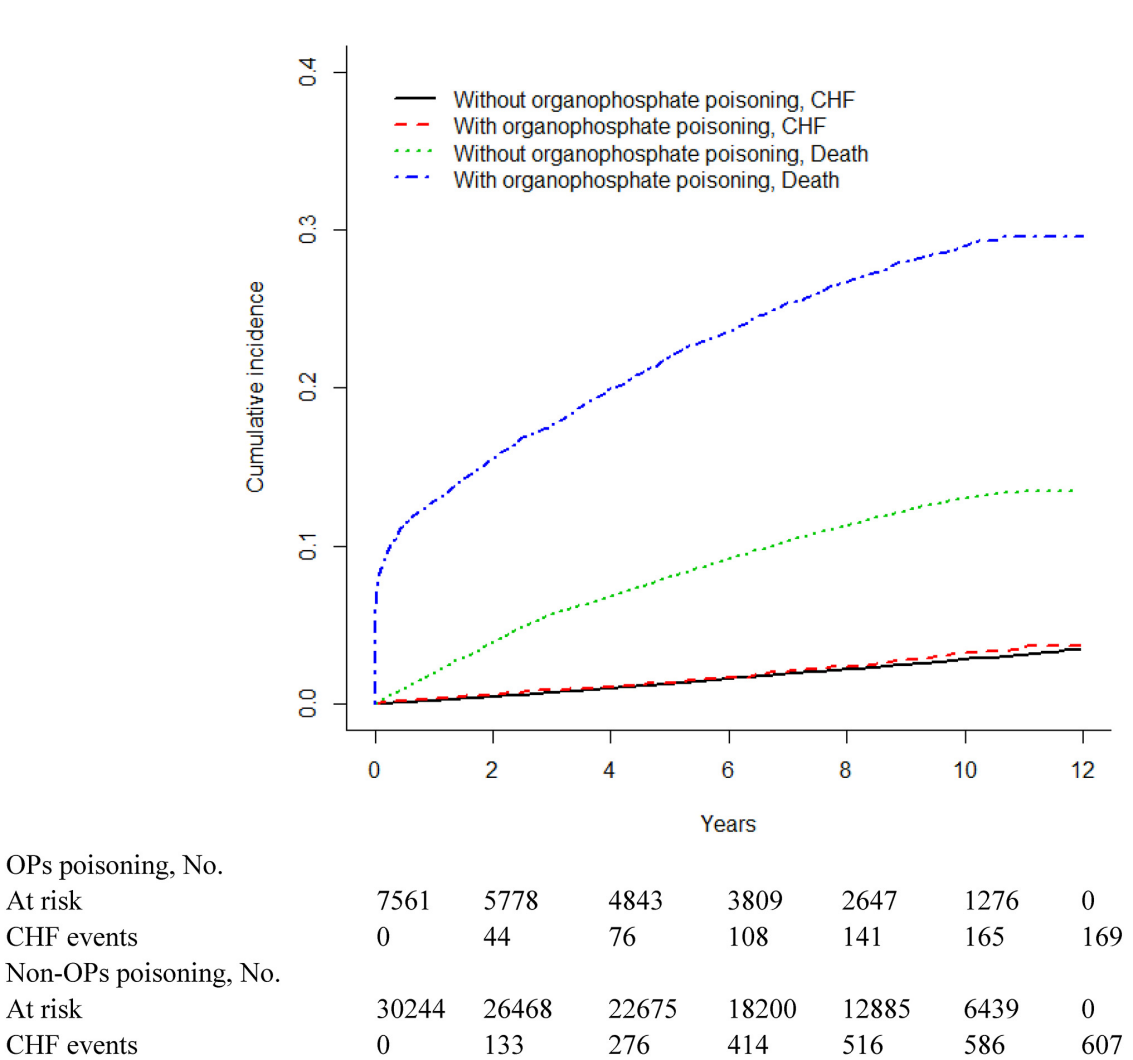
Cumulative incidence of congestive heart failure (CHF) compared between with and without organophosphate poisoning.

Figs 1–3 show the cumulative incidence curve of arrhythmia, CAD and CHF for the 2 cohorts after accounting for death as the competing risk indicated that the incidence of arrhythmia, CAD and CHF was higher among OPs poisoning patients than among non-OPs poisoning patients. Table 6 showed the survival analysis inference for each cohort. The 3-year and 5-year cumulative incidence rates were both higher among OPs poisoning patients than that among non-OPs poisoning patients.

**Table 6:** Cumulative incidence rates among different period.

	OPs poisoning			
	No		Yes	
Follow-up duration, year	%	95% CI	%	95% CI
Arrhythmia				
3 year	0.90	(0.58–1.41)	1.74	(0.95–3.17)
5 year	1.63	(1.28–2.09)	2.93	(2.04–4.19)
CAD				
3 year	1.95	(1.42–2.67)	2.90	(1.94–4.32)
5 year	3.34	(2.78–4.02)	4.56	(3.52–5.89)
CHF				
3 year	0.74	(0.40–1.38)	1.02	(0.41–2.55)
5 year	1.34	(0.95–1.89)	1.57	(0.87–2.86)

## Discussion

Heart attack due to CAD and its related complications remains one of the most prevalent causes of death worldwide. Several risk factors were established or suggested after extensive evidenced-based studies [20]. The strongest predictors of 10-year risk are age, sex, race, total cholesterol, high-density lipoprotein cholesterol (HDL-C), blood pressure, blood-pressure treatment status, diabetes, and current smoking status. The identification of novel risk factors for CVD is critical to improve our understanding of disease biology and to prevent cardiovascular morbidities and mortality. In the population-based cohort study presented herein, we found that OPs poisoning is a significant risk factor for cardiovascular diseases such as arrhythmia, CAD, or CHF. To our knowledge, there are no published reports regarding the potential adverse effects on heart health in a large population-based cohort of acute OPs poisoning.

In this community-based cohort study, we found that the estimated incidence density rates of any type of arrhythmia in cases of OPs poisoning were 1.6 times that of non-OPs poisoning individuals after age, sex, and comorbidity conditions were adjusted, although cases of OPs poisoning were significantly associated with chronic diabetes mellitus, hypertension, hyperlipidemia and COPD. It is also surprising that the vulnerability of arrhythmia lasted for more than 6 years.

The primary mechanism of OPs toxicity has been well studied and is known as inhibition of the acetylcholinesterase (AChE) enzyme. AChE is found in synapses, where it degrades the neurotransmitter acetylcholine and produces choline and acetate, a reaction important for controlling the operation of cardiac muscles–is the representative system affected by OPs. In a study conducted by Jayasinghe and Pathirana [21], without significant residual, autonomic dysfunction of cardiovascular system were found in 66 cases from a cohort study after 6 weeks follow-up acute OPs poisoning. Other toxic effects, such as oxidative stress induced by acute OPs poisoning or continuous reduction in M2 auto-receptor system, might explain the long term adverse effects of OPs on heart rhythms [11, 22, 23].

Additionally, excessive acetylcholine has been noted to be a potential etiological factor for reducing the threshold of epinephrine-induced arrhythmias after OPs poisoning in human and animal studies [13, 24]. Allon et al. indicated that the effect of locally released acetylcholine on epinephrine-induced arrhythmias could last for 6 months, and this may partly explain the delayed mortality observed in OPs poisoning patients [13].

In this large population-based study, we also found a possible correlation between acute OPs poisoning and ischemic heart diseases. The estimated incidence density rates of cardiovascular diseases (CAD and CHF) in cases of OPs poisoning were 1.3 times higher than those of non-OPs poisoning. After adjustment for age, sex, and co-morbidity conditions, the risk of CAD in cases of OPs poisoning remained significant (adjusted HR = 0.96, 95% CI = 0.85–1.08), but not for CHF (adjusted HR = 0.91, 95% CI = 0.76–1.09). The vulnerability of CAD or CHF in cases of OPs poisoning were also noted to last for 3 years, but not longer than arrhythmia. Additional, different mechanisms should be considered.

In ischemic heart disease, several risk factors have been studied and elucidated. Chronic disorders, such as diabetes mellitus, hypertension or hyperlipidemia, were considered to be modifiable factors for CAD [20]. In our cohort, acute OPs poisoning patients were noted to be associated with a higher rate of these chronic disorders and may compromise the cardiac effects of OPs after more than 3 years have passed since poisoning. OPs can lead to long-term inhibition of AChE and consumption of paraoxonase (PON1) by at least two mechanisms: first, plasma AChE binds OPs poisons; second, OPs are bioactivated to highly toxic oxon forms by cytochrome P450s, which is followed by destruction by hydrolysis by PON1 to harmless products [25]. PON1 may confer protection against damage of vessel walls by antioxidation and by destroying oxidation products [26]. Both the peroxidation of LDLs and secondary inflammatory responses are key steps in the initiation of atherogenesis [27]. Atherogenesis is the major cause of CAD and CHF. Thus, a decrease of PON1 activity caused by detoxification OPs is implicated in the pathogenesis of atherosclerosis and CVD [28].

It is also worth noting that the effect of OPs poisoning on CVD appears to be significant among relatively young populations. People who were exposed to OPs poisoning at 49 years of age or younger had a 1.48–3.16 times higher risk of CVD than those who were not exposed, while the hazards were not significantly different between individuals with and without OPs poisoning in individuals aged 50 years old or older. This is not unexpected because relatively older age (male > 45, female > 55) is a conventional risk factor for atherosclerotic cardiovascular disease, as stated in the American College of Cardiology and the World Heart Federation 2013 guidelines [20]. Thus, in older patients, the effect of OPs poisoning on CVDs could be masked by the homogeneous condition between the two groups. However, the information obtained from the large population-based cohort is important for the prevention of cardiac diseases in younger patients who survived acute OPs poisoning.

The CVD is the leading cause of death worldwide and with sophisticated mechanisms. Our findings suggest that OPs poisoning is another factor associated with the CVD risk. The CVD risk has been associated with traditional lifestyle factors of tobacco use, unhealthy diet and obesity, physical inactivity and harmful use of alcohol. It may be critical for clinical implication for patients with OPs exposure on the management of risk factors to prevent CVD. This may be particularly important for agriculture population at higher risk of OPs exposure.

The main strength of the present study is its population-based design and its generalizability. However, several limitations should be considered. First, administrative database studies are potentially prone to errors arising from the diagnostic code. The ICD-9-CM code 989.3 describes the toxic effects of organophosphate and carbamate. Carbamate poisoning shares clinical presentations of organophosphate poisoning with a shorter course due to its reversible inhibition of acetylcholinesterases. Delayed neuropathy due to carbamate intoxication is very rare and should always be disregarded [29], and the same is true of its late cardiac effects. The relative risk of long term cardiovascular diseases of OPs poisoning may be greater if the carbamate poisoning cases were excluded. The late cardiovascular complications of the OP poisoning may also be due to the continued occupational exposure to the low-level OP poisoning [30, 31]. Mills et al. [32] have observed little evidence of increased risk of cardiovascular complications associated with the occupational use of pesticides. Second, information on the lifestyle and behavior of patients is lacking in the NHIRD; thus, it was impossible to adjust for health- and behavior-related factors such as smoking, alcohol consumption, dietary habits, exercise, physical activity level, socioeconomic status, and body mass index, which are all potential confounding factors. Third, there are various types of organophosphates with different potencies and doses involved in toxic exposure among the patients, which may cause variable results. Fourth, habits of smoking, hypertension, high blood cholesterol, DM, obese and positive family history are noted to be major risk factors of CVD. COPD is a newly suggested to be a modifiable risk factor of CVD. In OPs cases, aspiration pneumonia was frequently noted to be a complication. So, in study of the possible correlation between OPs intoxication and CVD development, we used these 4 diseases as comorbidity. As we know, several factors, individual or combined, might contribute to these 4 diseases and not suitable to put together in the manuscript.

## Conclusions

In summary, this population-based retrospective cohort study has demonstrated a significant relationship between acute OPs poisoning and the risk of developing CVDs, which could persist for over 6 years, even for those with comorbidity of diabetes mellitus, hypertension and hyperlipidemia. Acetylcholine accumulation in nerve endings might be associated with the persistent cardiac injury for patients with OPs poisoning. The OPs are widely used in farms, industry and by consumers, and may cause neurological sequalae of acute poisoning, leading to cardiovascular impairment. Additional supportive measurements are essential in reducing the risk of heart disease. Furthermore, future studies are needed to investigate the chronic risk sequelae and possible mechanisms not only for heart diseases but also other organs associated with OPs poisoning.
